# An Unusual Case of Chylous Ascites

**DOI:** 10.7759/cureus.76018

**Published:** 2024-12-19

**Authors:** Thanda Aung, Mia Celestin, Sherona Bau, Sammy Saab

**Affiliations:** 1 Rheumatology, University of California Los Angeles David Geffen School of Medicine, Los Angeles, USA; 2 Hepatology, University of California Los Angeles David Geffen School of Medicine, Los Angeles, USA

**Keywords:** chylous ascites, complicated sclerosing mesenteritis, ig g4, igg4-related disease, pancreatic tumor

## Abstract

Chylous ascites occur when the lymphatic flow is blocked or disrupted, causing a leakage of fluid into the peritoneal space. It can be caused by a number of etiologies and identifying the exact cause can be challenging. We present the case of a 77-year-old man who presented with chylous ascites. An abdominal computed tomography scan revealed an abnormal spiculated mass and lymph nodes in the central mesentery encasing the pancreas, raising suspicion of pancreatic malignancy. A subsequent mesenteric biopsy indicated sclerosing mesenteritis characterized by dense fibrous tissue and positive Immunoglobulin G4 (IgG4) staining, leading to a final diagnosis of IgG4-related disease (IgG4-RD). The patient responded positively to a treatment regimen that included systemic steroids and rituximab. This case, with its atypical presentation of IgG4-RD, contributes to our understanding of this rare disease, providing valuable insights for future diagnosis and treatment.

## Introduction

Chylous ascites are triglyceride-rich peritoneal fluids in the abdominal cavity with a milky-white appearance. The causes of chylous ascites can vary based on the geographical location. In developed countries, likely causes include malignancy and cirrhosis, whereas in developing countries, infections such as tuberculosis or filariasis are high on the list of differential diagnoses [1]. In some instances, a tissue biopsy may be necessary. 

Immunoglobulin G4-related disease (IgG4-RD) is a rare and complex multi-organ, immune-mediated, fibro-inflammatory condition characterized by tumor-like lesions, elevated serum IgG4 levels, and infiltration of IgG4-positive plasma cells [2,3]. Typical presentations of IgG4-RD include major salivary lacrimal gland enlargement, orbital disease, autoimmune pancreatitis, retroperitoneal fibrosis, and tubulointerstitial nephritis [4]. Chylous ascites is an uncommon clinical presentation related to IgG4-RD. 

We describe a patient whose initial presentation of IgG4-RD was chylous ascites. Reliance on versatile tools was essential to confirm the diagnosis and treatment. 

## Case presentation

We describe a 77-year-old man who sought medical care for a few weeks' history of abdominal pain and distention. He denied weight loss, diarrhea, and rectal bleeding but reported sicca and fatigue. His past medical history included high blood pressure, coronary artery disease, and myocardial infarction. His physical examination was notable for significant abdominal distension. He denied a family history of malignancy or autoimmune diseases and denied any recent travel history. He underwent an abdominal ultrasound as a part of the evaluation for his abdominal distension and pain and it revealed a pancreatic mass measuring 2x1x0.5 cm. 

The initial labs, including a comprehensive metabolic panel (CMP) and complete blood count (CBC), were unremarkable except for mild normocytic anemia with hemoglobin 10.8 g/dL (reference range 13.5-17.1 g/dL) (Table 1).

**Table 1:** Results of the laboratory tests

Pertinent parameters	Patient's values	Reference range
Sodium (mmol/L)	136	135-146
Potassium (mmol/L)	4.3	3.6-5.3
Chloride (mmol/L)	100	96-106
Total CO_2_ (mmol/L)	23	20-30
Anion gap (mmol/L)	13	8.0-19.0
Glucose (mg/dL)	106	65-99
Estimated glomerular filtration rate or eGFR (mL/min/1.73m^2^)	63	>90
Urea nitrogen (mg/dL)	21	7.0-22.0
Calcium (mg/dL)	8.9	8.6 10.4
Total protein (g/dL)	8	6.1-8.2
Albumin (g/dL)	3.7	3.9-5.0
Bilirubin, Total (mg/dL)	0.2	0.1-1.2
Alkaline phosphatase (U/L)	85	31-113
Aspartate aminotransferase (U/L)	18	13-62
Alanine aminotransferase (U/L)	23	8.0-70.0
White blood cell count (x10^3^/µL)	7.46	4.16-9.95
Red blood cell count (x10^6^/µL)	4.34	4.41-5.95
Hemoglobin (g/dL)	10.8	13.5-17.1
Hematocrit (%)	39.1	38.5-52.0
Mean corpuscular volume (fL)	90.1	79.3- 98.6
Mean corpuscular hemoglobin (pg)	28.6	26.4-33.4
Mean corpuscular hemoglobin concentration (g/dL)	31.7	31.5-35.5
Red cell distribution width - SD (fL)	60.3	36.9-48.3
Red cell distribution width - CV (%)	18.4	11.1-15.5
Platelet Count, auto (10^3^/µL)	270	143-398
Mean platelet volume (fL)	9.5	9.3-13.0
Absolute nucleated red blood cell count (x10^3^/µL)	0.00	0.00-0.00
Neutrophil antibodies (x10^3^/µL)	5.73	1.80-6.90
Absolute lymphocyte count (x10^3^/µL)	0.49	1.30-3.40
Absolute monocyte count (x10^3^/µL)	0.80	0.20-0.80
Absolute eosinophil count (x10^3^/µL)	0.33	0.00-0.50
Absolute basophil count (x10^3^/µL)	0.05	0.00-0.10
Absolute immature granulocyte count (x10^3^/µL)	0.06	0.00 - 0.04

The results of the ascitic fluid analysis were remarkable for a high triglyceride level at 3740 mg/dL (reference value <150 mg/dL) and protein at 7.2 gm/dL (reference value <2.5 gm/dL), and no malignant cells were identified (Table 2).

**Table 2:** Paracentesis fluid analysis

Pertinent parameters	Patient's values	Reference range
Triglyceride (mg/dL)	3740	<150
Protein (gm/dL)	7.2	<2.5

**Figure 1:**
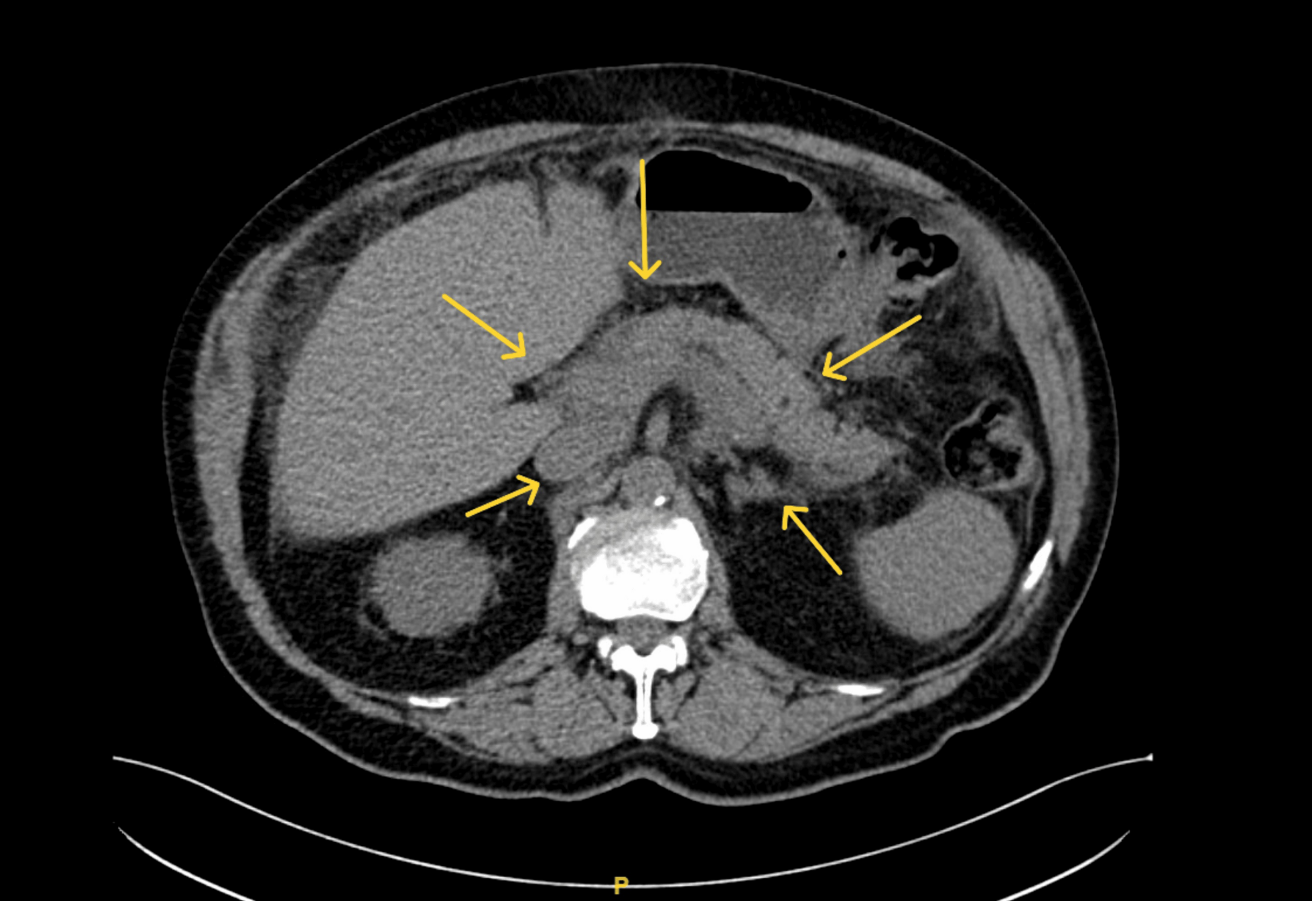
Computer tomography of the abdomen Arrows pointing at homogeneously-enhancing pancreatic parenchyma.

Imaging with CT and MR cholangiopancreatography (MRCP) was done. The CT of the abdomen with contrast showed an abnormal spiculated mass, conglomerate lymph nodes in the right central mesentery, and a lobar, lower attenuating soft tissue partially encasing the pancreas, thought to be a pancreatic malignancy (Figure 1).

**Figure 2:**
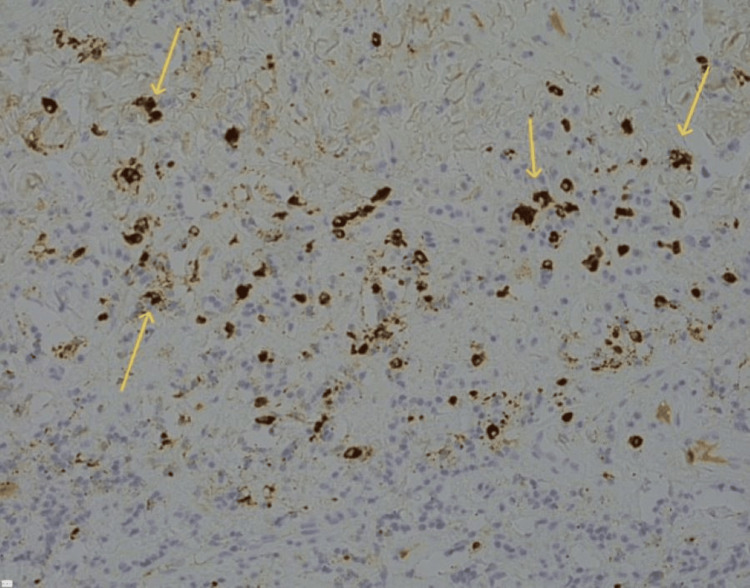
Immunohistochemical stain for IgG4 with hematoxylin counterstain Arrows pointing at the brown plasma cells which are positive for IgG4.

The results of MRCP showed retroperitoneal infiltration of the head/neck and pancreas extending to the mesenteric root, which presented nodules and ascites. A mesenteric biopsy revealed sclerosing mesenteritis, dense fibrous tissue with a lymphoplasmacytic infiltrate, and increased plasma cell concentration. IgG4 staining confirmed IgG4-sclerosing disease (Figure 2). 

Other rheumatology disease work-ups showed antinuclear antibodies (+ANA) but no evidence for lupus, Sjogren's, or connective tissue diseases. Vasculitis work-up was negative and the sarcoidosis panel was also normal. The inflammatory marker CRP was normal; however, the patient had low complement 4 (C4) levels at <2 mg/dL (reference range 10-40 mg/dL) and elevated total IgG at 1,638 mg/dL (reference range 700-1600 mg/dL), IgG1 at 1,379 mg/dL (reference range 240-1118 mg/dL), and erythrocyte sedimentation rate (ESR) at 90 mm/hr (reference value <=12 mm/hr). IgG4, however, was normal (Table 3).

**Table 3:** Results of laboratory tests ANA: Antinuclear antibody; dsDNA Ab EIA: double-stranded DNA antibody enzyme immunoassay; nDNA Ab IFA: native DNA indirect fluorescent antibody; SSA: Sjögren's-syndrome-related antigen A; SSB: Sjögren's-syndrome-related antigen B; Cyclic citrulline Ab IgG: Cyclic citrulline antibody Immunoglobulin G; C-ANCA: Cytoplasmic antineutrophil cytoplasmic antibody; P- ANCA: Perinuclear antineutrophil cytoplasmic antibody, Protein 3 Ab: Protein 3 antibody; Myeloperoxidase Ab: Myeloperoxidase antibody; C3: Complement 3; C4: Complement 4; ACE: Angiotensin-converting enzyme; ESR:  Erythrocyte sedimentation rate; IgG: Immunoglobulin G; IgG1: Immunoglobulin G1, IgG2: Immunoglobulin G2, IgG3: Immunoglobulin G3, IgG4: Immunoglobulin G4.

Pertinent parameters	Patient's values	Reference range
ANA Ab Titer	1:640 (homogeneous)	<1:40
dsDNA Ab EIA (IU/mL)	<=200	<=200
nDNA (Crithidia) Ab IFA (titer)	<1:10	<1:10
SSA (U)	<20	<20
SSB (U)	<20	<20
Rheumatoid factor (IU/mL)	<10	<14
Cyclic citrulline Ab IgG (units)	2	0-19
C-ANCA (titer)	<1:20	<1:20
P-ANCA (titer)	<1:20	<1:20
Protein-3 Ab (CU)	<20.0	<20.0
Myeloperoxidase Ab (CU)	<20.0	<20.0
C3 (mg/dL)	105	86-175
C4 (mg/dL)	<2	10.0-40.0
ACE (U/L)	<10	16 - 85
Cryocrit	Negative	Negative
ESR (mm/hr)	90	<=12
C-reactive protein (mg/dL)	0.7	<0.8
IgG total (mg/dL)	1638	700-1600
IgG1 (mg/dL)	1379	240-1118
IgG2 (mg/dL)	252	124-549
IgG3 (mg/dL)	53	21-134
IgG4 (mg/dL)	70	1-123

Immunofixation and serum work-up by hematology revealed a persistent monoclonal band of IgM, elevated kappa and lambda light chains, and IgG levels. The patient later underwent a bone marrow biopsy, which was negative for myeloid and lymphoproliferative disorders. Extensive infection disease work-up was negative for fungal infections, mycobacteria infection, hepatitis B, hepatitis C, and filariasis. 

He was treated with diuretics and high-dose steroids (1 mg/kg body weight). Due to persistent ascites and persistent elevation of sedimentation rate after six weeks of steroid therapy, rituximab infusion was added to his regimen with two doses of 1000 mg administered two weeks apart. Four months after the initial rituximab therapy, there was significant clinical improvement with the resolution of ascites, and serologically, IgG1 level and sedimentation rate were within normal limits. He continued rituximab as maintenance therapy with two doses of 1000 mg administered two weeks apart every six months. Systemic oral steroids were tapered down and stopped one year after his initial treatment. Currently, he is asymptomatic, and follow-up imaging shows a reduction in the size of the pancreatic mass, and there are no more ascites. 

## Discussion

The typical marker for IgG4-RD is elevated IgG4 levels. However, this patient presented with normal IgG4 levels but persistently elevated IgG1 and ESR. While elevated serum IgG4 levels are a characteristic feature of IgG4-RD, the diagnostic utility of IgG4 levels alone is limited. Several studies have reported cases of biopsy-proven IgG4-RD with normal serum IgG4 levels (1-123 mg/dL) [5,6]. The sensitivity of elevated serum IgG4 for diagnosing IgG4-RD ranges from 60% to 80% [3]. Therefore, a normal IgG4 level does not exclude the diagnosis of IgG4-RD, and histopathological examination remains the gold standard for diagnosis [5]. Elevated IgG1 levels have been reported in various inflammatory and autoimmune conditions, but their significance in IgG4-RD needs to be better established [7]. 

The management of IgG4-RD induction therapy typically consists of systemic steroids and immune suppressant therapy as steroid-sparing agents. For systemic steroids, prednisone is generally given at 0.5-1 mg/kg and tapered off according to the disease activity. The first-line immune suppressant therapy for induction remission is rituximab, an anti-CD-20 monoclonal antibody [8]. If rituximab is unavailable or contraindicated, second-line agents like azathioprine or mycophenolate can be used. There are studies where both agents can be helpful in patients with IgG4-RD presenting with autoimmune pancreatitis (AIP) or sclerosing cholangitis. Still, there are no head-to-head studies to compare their efficacy against rituximab [9-11]. Case reports suggest that abatacept, an Immunosuppressive cytotoxic T lymphocyte-associated antigen-4 immunoglobulin fusion proteins (CTLA4-Ig), and dupilumab, a monoclonal antibody against interleukin (IL) 4 receptor, may both be effective in patients with IgG4-RD, but further studies must be done to determine their efficacy [12-14]. Typically, the disease responds well if treated before any detrimental organ damage [8]. Maintenance therapy with agents such as rituximab or other immune suppressants such as azathioprine or mycophenolate is essential to prevent disease relapse. The average time for relapse with rituximab was 41 months (about three and a half years) vs 21 months (about two years) without it [15]. While treatment is essential for IgG4-RD, relapses often occur once treatment is discontinued. The therapies mentioned above are not a cure for the disease. A study found that around 30% of patients treated with rituximab relapsed once treatment was stopped [16,17]. This prognosis shows that further research must be done to find long-lasting therapies for this disease. 

## Conclusions

IgG4-RD can affect multiple organs and often results in the formation of secondary mass-like tumors. It can frequently mimic various malignancies, making accurate diagnosis challenging. Not all cases of IgG4-RD have elevated IgG4 levels and this diversity of presentation further increases the difficulty of diagnosing the condition. Due to the heterogeneous manifestations of the disease, histopathology remains the gold standard for diagnosis, as seen in our case when the diagnosis was confirmed via biopsy. While treatment may vary from case to case, quick and effective treatment is essential, and systemic steroids and rituximab have proven beneficial. Our case explores an unusual scenario of chylous ascites as a rare presentation of IgG4-RD. As this is atypical, further studies are required to better understand the pathogenesis, disease outcome, prognosis, and other treatment options for this condition. 
